# A DNA barcode reference library of Croatian mosquitoes (Diptera: Culicidae): implications for identification and delimitation of species, with notes on the distribution of potential vector species

**DOI:** 10.1186/s13071-024-06291-9

**Published:** 2024-05-11

**Authors:** Nataša Bušić, Ana Klobučar, Nediljko Landeka, Toni Žitko, Goran Vignjević, Nataša Turić, Mirta Sudarić Bogojević, Enrih Merdić, Mladen Kučinić, Branka Bruvo Mađarić

**Affiliations:** 1 Department of Biology, Josip Juraj Strossmayer University of Osijek, Osijek, Croatia; 2 Andrija Štampar Teaching Institute of Public Health, Zagreb, Croatia; 3 Teaching Institute for Public Health of the Istrian County, Pula, Croatia; 4 Teaching Institute for Public Health, Split-Dalmatia County, Split, Croatia; 5 Teaching Institute for Public Health of the Osijek-Baranja County, Osijek, Croatia; 6 Faculty of Science, Department of Biology, University of Zagreb, Zagreb, Croatia; 7 Department of Molecular Biology, Ruđer Bošković Institute, Zagreb, Croatia

**Keywords:** Culicidae., Cytochrome c oxidase I (COI)., Internal transcribed spacer 2 (ITS2)., Species identification., Species delimitation., Cryptic species., Species complex., Invasive species.

## Abstract

**Background:**

Mosquitoes pose a risk to human health worldwide, and correct species identification and detection of cryptic species are the most important keys for surveillance and control of mosquito vectors. In addition to traditional identification based on morphology, DNA barcoding has recently been widely used as a complementary tool for reliable identification of mosquito species. The main objective of this study was to create a reference DNA barcode library for the Croatian mosquito fauna, which should contribute to more accurate and faster identification of species, including cryptic species, and recognition of relevant vector species.

**Methods:**

Sampling was carried out in three biogeographical regions of Croatia over six years (2017–2022). The mosquitoes were morphologically identified; molecular identification was based on the standard barcoding region of the mitochondrial COI gene and the nuclear ITS2 region, the latter to identify species within the *Anopheles maculipennis* complex. The BIN-RESL algorithm assigned the COI sequences to the corresponding BINs (Barcode Index Number clusters) in BOLD, i.e. to putative MOTUs (Molecular Operational Taxonomic Units). The bPTP and ASAP species delimitation methods were applied to the genus datasets in order to verify/confirm the assignment of specimens to specific MOTUs.

**Results:**

A total of 405 mosquito specimens belonging to six genera and 30 morphospecies were collected and processed. Species delimitation methods assigned the samples to 31 (BIN-RESL), 30 (bPTP) and 28 (ASAP) MOTUs, with most delimited MOTUs matching the morphological identification. Some species of the genera *Culex*, *Aedes* and *Anopheles* were assigned to the same MOTUs, especially species that are difficult to distinguish morphologically and/or represent species complexes. In total, COI barcode sequences for 34 mosquito species and ITS2 sequences for three species of the genus *Anopheles* were added to the mosquito sequence database for Croatia, including one individual from the Intrudens Group, which represents a new record for the Croatian mosquito fauna.

**Conclusion:**

We present the results of the first comprehensive study combining morphological and molecular identification of most mosquito species present in Croatia, including several invasive and vector species. With the exception of some closely related species, this study confirmed that DNA barcoding based on COI provides a reliable basis for the identification of mosquito species in Croatia.

**Graphical Abstract:**

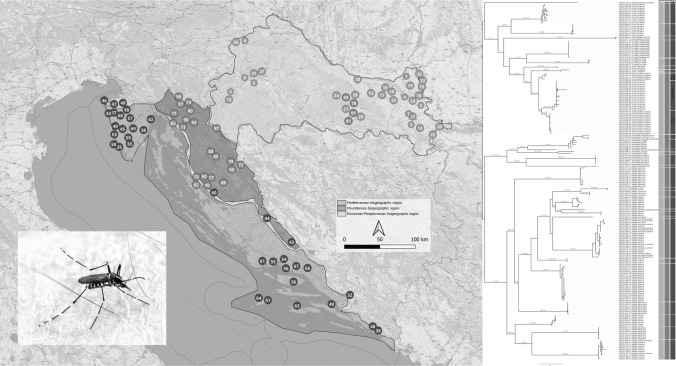

**Supplementary Information:**

The online version contains supplementary material available at 10.1186/s13071-024-06291-9.

## Background

Mosquitoes are one of the most studied groups of insects in the world, mainly because of their medical and veterinary role as vectors of pathogens causing millions of deaths per year.. Although most data on mosquito-borne diseases mainly refer to tropical and subtropical countries, quite a few cases of such infections have also recently been recorded in Europe [1–4]. Due to increasing trade and travel, invasive mosquito species are introduced and spread very easily [5–7], such as *Aedes albopictus* and *Aedes aegypti,* which are vectors of many viruses that cause various infectious diseases (e.g. dengue, chikungunya, yellow fever, Japanese encephalitis, West Nile and Zika viruses) [8–10]. In Croatia, well established populations of *Aedes albopictus* are present [11], while *Aedes aegypti* has not yet been recorded. In 2010, autochthonous cases of dengue fever caused by the dengue virus were registered for the first time in Croatia [12]. Also, several smaller dengue epidemics were continuously registered in Europe, with a number of autochthonous cases in France, Spain and Italy [13–15]. Although invasive species pose a serious medical problem, native mosquito species such as those of the genus *Culex* should not be ignored. This has been demonstrated by recent cases of West Nile virus (WNV) outbreaks in Romania, Spain, the Netherlands, Italy, Hungary, Germany, and Serbia [3, 16–18]. In Croatia, the first clinical cases of WNV infection were reported in 2012 [19], associated with *Culex pipiens* complex, and possibly some other species [20]. After 2012, WNV infections occurred continuously in Croatia [21]. The main vector of Usutu virus (USUV) infection is *Culex pipiens*, although the virus has also been found in several other species [22, 23]. Recent studies show that USUV has become endemic in northwestern Croatia [24]. Malaria used to be a prevalent disease in Europe, but currently only imported cases are reported; no deaths occurred following autochthonous infections in 2000–2019 [25]. However, numbers of imported malaria cases in Europe have increased, leading to the re-emergence of indigenous cases in Greece, Spain, Italy, and France [26–29]. Within the *Anopheles maculipennis* complex, several species are considered vectors of malaria parasites in Europe: *Anopheles atroparvus, Anopheles labranchiae, Anopheles messeae* and *Anopheles sacharovi* [30, 31].

The correct identification of mosquitoes is an important part of implementing effective vector management strategies. Conventional identification with dichotomous keys is essential, but has many shortcomings and is not always sufficient to identify mosquito specimens. Important features required for accurate morphological identification of mosquitoes often fall off or are damaged during sampling (scales, legs, wings), or these defining differences are only visible at a certain stage of development or are related to sex. In addition, mosquitoes often occur in complexes of closely related species, and morphological identification proved to be insufficient in most of these cases [32].

Molecular identification by DNA barcoding is an accurate method of species identification, independent of the developmental stage and condition of the specimens examined [33–35]. Many studies have confirmed that the standard mitochondrial COI barcoding region is a suitable marker for identification of mosquito species and recognition of cryptic species [36–41]. Nevertheless, for several genera and species complexes (*Culex, Aedes* and *Anopheles*), the use of additional molecular markers is necessary to increase identification accuracy and to distinguish between closely related species, forms and hybrids. For example, ITS2 (nuclear ribosomal internal transciber spacer 2), Ace2 gene and polymorphisms of various microsatellite loci (e.g. CQ11) are now routinely used [42–47]. However, despite major advances in molecular methods, accurate identification requires a multidisciplinary approach to taxonomy that includes morphological, molecular, distributional and ecological data [36, 48].

To date, 52 species of mosquitoes have been recorded in Croatia, two of which are invasive: *Aedes albopictus* and *Aedes japonicus* [11]. Traditionally, the majority of research has been based on morphological identification, but several recently published papers have used molecular methods to confirm the presence of certain species. By these, *Cx. torrentium* was proven for the first time to belong to the mosquito fauna of Croatia in 2018 [49], and the presence of several other species was confirmed for certain regions of Croatia [50, 51]. However, no systematic survey has been conducted to date that would provide an overview of the barcodes of the Croatian mosquito fauna, and there is also very little data for neighbour countries [52, 53]. The importance of such data lies in the potential presence and spread of new invasive species that are potential arbovirus vectors, such as *Aedes koreicus*, which is currently established in surrounding countries but has not yet been recorded in Croatia [54, 55]. Recent DNA barcoding studies in Europe [56–64] established national DNA barcode libraries for several countries, and resulted in new species findings and confirmation of unrecorded species in certain areas, as well as identification of cryptic taxonomic units [65]. Nevertheless, the number of barcode sequences of European mosquitoes with accurate country and species designation is still limited and accounts for less than 10% of all Culicidae records in BOLD (accessed December 15, 2023).

The aim of this study was to create a DNA barcode library for the Croatian mosquito fauna and to gain insight into the genetic diversity and geographical distribution of mosquito species through DNA barcoding. The data obtained will contribute to public databases (BOLD, NCBI GenBank) and help create a platform for easier, faster and more accurate identification of mosquitoes of the Croatian fauna. The results will also serve as a basis for projects on the surveillance of invasive and vector mosquitoes and control of mosquito-borne diseases in the studied region.

## Methods

### Study area

With its geographical location in Central Europe and the Mediterranean region, Croatia is one of the "hotspots" of European biodiversity. The high level of biodiversity in Croatia is a consequence of the diverse composition of habitats, climatic and hydrological characteristics and the complex geological history of the region.

**Fig. 1:**
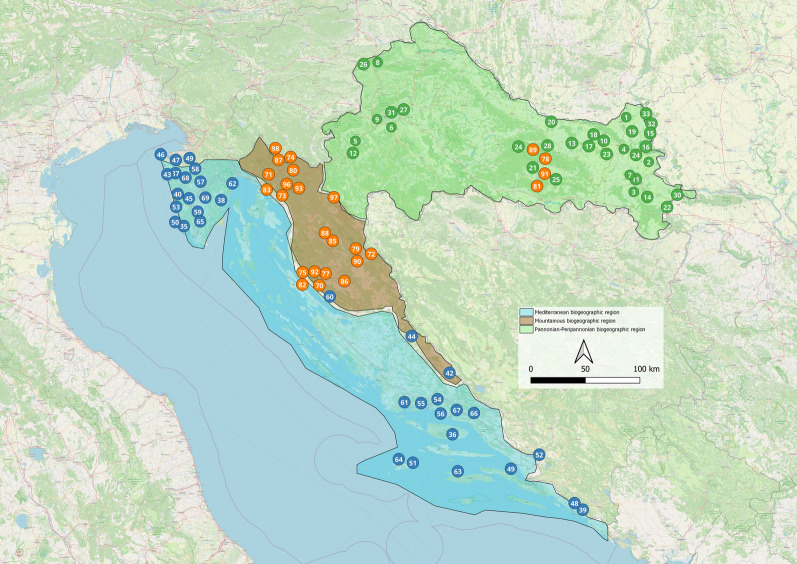
Sampling localities in ​​three biogeographic regions of Croatia: Pannonian-Peripannonian region—green dots, Mediterranean region—blue dots, and mountainous region—orange dots; high-altitude localities in the Pannonian-Peripannonian region are also marked with orange dots. Some localities may represent multiple sampling sites (details for each sample are available in the public BOLD dataset DS-CROCU2)

According to Bertić et al. [66], Croatia was divided into three biogeographic regions for the purpose of this study:the Pannonian-Peripannonian region in the north and east, the central mountainous region in the middle and the Mediterranean region in the south (Fig. 1). From 2017 to 2022, 50 sites were sampled in the Pannonian-Peripannonian region, 38 in the central mountainous region and 52 in the Mediterranean region of Croatia (Fig. 1; Additional file: Table S1).

### Mosquito collection and a priori morphological identification

Adult mosquitoes were caught with CDC traps baited with CO_2_, BG-Sentinel traps baited with BG-Lure and CO_2_ and human landing catch (HLC) method. Larvae were caught individually using a dipper. The mosquitoes were morphologically identified using the identification keys of Becker et al. [67]. All specimens were stored in 96% ethanol at -20° C. The vouchers of the barcoded specimens are kept at the Josip Juraj Strossmayer University of Osijek, Department of Biology, and at the Andrija Štampar Teaching Institute of Public Health in Zagreb.

### DNA extraction and amplification

DNA was extracted from single legs of adult specimens and from entire larvae using the GenEluteTM Mammalian Genomic DNA Miniprep Kit (Sigma, Taufkirchen, Germany). The protocol for rodent tail preparation as provided with the kit was followed with slight modifications (incubation in proteinase K overnight; final DNA elution in 100 µl elution solution).

For all samples, the standard barcoding region of the mitochondrial COI gene [34] was amplified using the universal primers LCO1490 and HCO2198 [68]. For certain species complexes, the COI gene marker does not provide sufficient resolution, so other genomic regions were used. For the identification of species within the *Anopheles maculipennis* complex, the ITS2 region was amplified with the primers 5.8S (forward) and 28S (reverse) [69]. The amplification mixtures and PCR reaction conditions for COI and ITS2 are described in Bušić et al. [50]. The PCR products were enzymatically purified using the ExoI-rSAP system (NEB, Ipswich, MA, USA) according to the manufacturer's protocol and bidirectionally sequenced at Macrogen Inc. (Amsterdam, The Netherlands) using the amplification primers.

### Data analysis

The sequences of the COI and ITS2 regions were verified and edited in Geneious 8.1.4. (https://www.geneious.com) and subsequently deposited in the NCBI GenBank and BOLD databases (GenBank accession numbers PP694665-PP694812, BOLD ID numbers in Additional file 1: Table S1; additional information available in public BOLD dataset DS-CROCU2). The percentage identity of the newly obtained COI and ITS2 sequences with the records in the GenBank database was checked with the BLAST tool using the Megablast algorithm (https://blast.ncbi.nlm.nih.gov/Blast.cgi; accessed December 15, 2023). The BOLD identification tool (http://www.boldsystems.org/index.php/IDS_OpenIdEngine; accessed December 15, 2023) was used to compare COI barcode sequences amplified from our samples with publicly available data in the BOLD database. Available published COI sequences of conspecific and congeneric mosquito specimens were selected from the BOLD database of public records (accessed December 15, 2023) and used for subsequent analyses.

COI and ITS2 sequences were analysed in datasets corresponding to specific genera. Multiple sequence alignments were performed with MAFFT version 7 using the "auto" strategy [70] (https://mafft.cbrc.jp/alignment/server/index.html; final alignments in Additional file 2: Dataset S1). Intraspecific and interspecific p-distances were calculated in MEGA 7.0.25 [71]. Maximum likelihood (ML) trees were generated on the PhyML 3.0 web server [72] (http://www.atgc-montpellier.fr/phyml/), with automatic model selection by SMS (Smart Model Selection; model determined by the AIC selection criterion) [73] and aLRT SH-like support [74].

Species delimitation methods (SDMs) bPTP [75] (https://species.h-its.org/ptp/) and ASAP [76] (https://bioinfo.mnhn.fr/abi/public/asap/asapweb.html) were applied to genus datasets to verify/confirm the assignment of specimens to specific Molecular Operational Taxonomic Units (MOTUs). As input for bPTP, the inferred ML tree was used, while for the ASAP method, the MAFFT alignment was used. In addition, the BIN-RESL algorithm (Barcode Index Number system in BOLD) [77] assigned the sequences to the corresponding BINs in BOLD. The results of the SDMs are presented in a combined ML tree that includes all newly barcoded specimens. The tree was graphically processed in FigTree v.1.4.3. (http://tree.bio.ed.ac.uk/software/figtree/).

## Results

In this study, a total of 405 mosquito specimens were collected and processed; 181 specimens in the Pannonian-Peripannonian region, 122 specimens in the Mediterranean region and 102 specimens in the mountainous region of Croatia. Based on morphological identification, a total of 30 species previously recorded in Croatia were sampled, belonging to six genera (*Aedes*—16 species, *Anopheles*—5 species, *Culex*—5 species, *Culiseta*—2 species, *Coquillettidia*—1 species and *Orthopodomyia*—1 species) (Table 1). For some specimens, such as those of the *Aedes annulipes/cantans/excrucians* and *Aedes cinereus/rossicus* groups or some species complexes such as the *Culex pipiens* and *Anopheles maculipennis* complexes, it was not possible to determine morphologically to which species they belonged.

**Table 1:** Mean and maximum intraspecific distance (intra-SP) between individuals of the same BIN (species / MOTUs) and distance to nearest neighbour (NN) BIN in BOLD

BIN	Total BIN members	Taxa (this project)	Specimen count (this study)	Taxa included in the BIN (specimen count)	BIN mean intra-SP (%)	BIN max. intra-SP (%)	Distance to NN BIN (%)	NN BIN (taxa)
BOLD: AAA3748	1688	*Aedes punctor*	2	*Aedes punctor* (280); *Ae. hexodontus* (256); *Ae. abserratus* (125); *Ae. aboriginis* (12); *Aedes* sp. (13); *Ae. diantaeus* (9); *Ae. ventrovittis* (4); *Ae. nigripes* (3); *Ae. communis* (3); *Ae. pionips* (4); *Ae. fitchii* (1); *Ae. decticus* (1); *Ae. intrudens* (1); *Ae.* nr. *punctor* (1)	1.68	4.26	1.76	BOLD:AEI5390 (*Aedes* sp. HANKV_BC_Contig1)
BOLD: AAA4751	6020	*Culex pipiens* complex	17	*Culex quinquefasciatus* (3126); *Cx. pipiens* (2002); *Culex* sp. (29); *Cx. torrentium* (358); *Cx.* nr. *pipiens* (38); *Cx. pipiens* s.l. (275);	0.57	5.02	0.98	BOLD:AEW0334 (*Culex pipiens*)
BOLD: AAA5870	4104	*Aedes albopictus*	7	*Aedes albopictus* (4062); *Ae. aegypti* (7); *Ae. malayensis* (2); *Ae. flavopictus* (1); Culicidae sp. (5); *Aedes* sp. (2); *Culiseta annulata* (1)	0.49	3.56	3.88	BOLD:ACH4817 (*Aedes flavopictus*)
BOLD: AAA6148	613	*Aedes communis*	3	*Aedes communis* (338); *Ae. intrudens* (5); *Ae. punctor* (6); *Ae. sticticus* (4); *Ae. dorsalis* (1); *Ae. pullatus* (2)	0.53	3.38	3.45	BOLD:AAB6338 (*Aedes pionips*)
BOLD: AAA7067	5018	*Aedes vexans*	7	*Aedes vexans* (4881); *Ae.* nr. *vexans* (2) *Ae. sticticus* (2); *Ae. intrudens* (1); *Ae. vexans nipponii* (31); *Ae. vexans vexans* (6)	1.22	7.22	3.66	BOLD:ACZ5331 (JOMOS466-15 *Aedes* sp.)
BOLD: AAA9632	515	*Anopheles maculipennis* s.s	2	*Anopheles maculipennis* (323); *Anopheles* sp. MBI-36 (96); *An. maculipennis* s.s. (92); *An. messeae* (2); *An. maculipennis* s.l. (1); *An. melanoon* (1)	1.50	3.99	1.33	BOLD:ABY8239 (*Anopheles messeae/ daciae*)
		*Anopheles maculipennis* complex	1					
BOLD: AAB1098	1465	*Aedes annulipes*	1	*Aedes excrucians* (368); *Ae. cantans* (166); *Ae. annulipes* (95); *Ae. flavescens* (71); *Ae. stimulans* (58); *Ae. fitchii* (42); *Ae. euedes* (7); *Ae. riparius* (3); *Aedes* sp. (11); *Ae. rusticus* (1)	1.22	3.66	1.76%	BOLD:AAD4406 (SSKUA985-15 *Aedes* sp.)
		*Aedes cantans*	10					
		*Aedes excrucians/cantans*	1					
		*Aedes cantans/riparius*	1					
		*Aedes annulipes/cantans*	9					
BOLD: AAB2483	237	*Anopheles hyrcanus*	1	*Anopheles hyrcanus* (63); *An. pullus* (136); *An. pseudopictus* (37)	1.58	5.32	2.09	BOLD:AAA5339 (*Anopheles kweiyangensis*)
BOLD: AAB6945	48	*Culex territans*	7	*Culex impudicus* (26); *Cx. territans* (17); *Cx. pipiens* (2)	0.24	1.66	1.12	BOLD:AEE2449 (*Culex territans*)
BOLD: AAB7911	585	*Aedes caspius*	4	*Aedes caspius* (506); *Ae.* nr. *caspius* (23); *Ae. zammitii* (7); *Ae. dorsalis* (5); *Ae. geniculatus* (1); *Aedes* sp. (3); *Culex perexiguus* (1); *Anopheles atroparvus* (2); *Ae. detritus* (1)	1.15	4.14	1.13	BOLD:ACE6286 (*Aedes dorsalis*)
		*Aedes zammitii*	3					
BOLD: AAC5210	730	*Aedes japonicus*	11	*Aedes japonicus* (726); *Ae. bhutanensis* (2); *Ae. sierrensis* (1); *Ae. japonicus japonicus* (157)	0.65	3.92	7.56	BOLD:ACB6413 (*Aedes koreicus*)
BOLD: AAD6954	330	*Culiseta annulata*	4	*Culiseta annulata* (322); *Culiseta alaskaensis* (1); *Culiseta subochrea* (4)	0.20	2.08	2.56	BOLD:AAV9075 (*Culiseta subochrea*)
BOLD: AAE3979	44	*Anopheles claviger*	1	*Anopheles claviger* (6)	1.20	3.15	1.82	BOLD:AAM4220 (*Anopheles claviger*)
BOLD: AAF2904	318	*Aedes intrudens/diantaeus/pullatus*	1	*Aedes intrudens* (154); *Ae. diantaeus* (133); *Aedes* sp. (1); *Ae. communis* (1); *Ae. aurifer* (1); *Ae. pullatus* (1)	1.36	3.37	3.37	BOLD:AAU0369 (*Aedes aurifer*)
BOLD: AAI5767	72	*Culex hortensis*	11	*Culex hortensis* (70); *Cx. pipiens* (2)	0.84	1.96	6.40	BOLD:AAZ3152 (*Culex adairi*)
BOLD: AAJ7317	424	*Culex modestus*	2	*Culex modestus* (419); *Culex* sp. (3); *Anopheles* nr. *algeriensis* (1); *Cx. pipiens* (1)	1.12	3.55	1.41	BOLD:AEL0567 (*Culex modestus*)
BOLD: AAM2826	155	*Aedes detritus*	3	*Aedes detritus* (142); *Ae. pulcritarsis* (2); *Aedes* sp*.* (1); *Anopheles atroparvus* (1); *Culex torrentium* (1)	1.13	3.32	5.57	BOLD:AAC9531 (*Aedes* CNKOS142-14)
BOLD: AAM4220	115	*Anopheles claviger*	1	*Anopheles claviger* (87); *An. claviger* s.l. (25); *Coquillettidia richiardii* (2)	1.00	3.56	1.82	BOLD:AAE3979 (*Anopheles claviger*)
BOLD: AAM5033	59	*Aedes rusticus*	3	*Aedes rusticus* (56); *Ae. cantans* (1)	0.33	1.78	3.56	BOLD:AAD4355 (*Aedes provocans*)
BOLD: AAN1645	84	*Aedes pulcritarsis*	1	*Aedes pulcritarsis* (72);	1.10	2.89	5.14	BOLD:AAB7911 (*Aedes caspius*)
BOLD: AAN3326	92	*Anopheles plumbeus*	6	*Anopheles plumbeus* (90); *An. claviger* s.l. (1); *An. claviger* (1)	0.11	1.19	9.89	BOLD:AEA7027 (*Anopheles veruslanei*)
BOLD: AAP0901	314	*Culiseta longiareolata*	10	*Culiseta longiareolata* (302); *Cx. modestus* (1)	0.27	1.85	2.56	BOLD:AEG1924 (*Culiseta longiareolata*)
BOLD: AAP8897	98	*Aedes cinereus*	8	*Aedes cinereus* (78); *Aedes* sp. (8). *Ae. rossicus* (6); *Ae.* nr. *cinereus* (3)	0.65	3.16	1.43	BOLD:AAP8896 (*Aedes cinereus*)
		*Aedes rossicus*	1					
		*Aedes rossicus/cinereus*	2					
BOLD: AAR3271	82	*Anopheles algeriensis*	1	*Anopheles algeriensis* (68)	1.45	3.43	4.23	BOLD:ACR6429 (*Anopheles* sp. nr *algeriensis* MBI2015)
BOLD: AAS0072	180	*Coquillettidia richiardii*	2	*Coquillettidia richiardii* (179); *Aedes annulipes* (1)	0.57	2.17	6.04	BOLD:AAI1618 (*Coquillettidia perturbans*)
BOLD: AAW9535	3	*Orthopodomyia pulcripalpis*	1	*Orthopodomyia pulcripalpis* (1)	0.00	0.00	4.97	BOLD:AAW9539 (*Orthopodomyia alba*)
BOLD: ABY8239	698	*Anopheles messeae*	1	*Anopheles messeae* (228); *An. daciae* (90); *An. daciae* sp. inq. (376); *Anopheles* sp. (2); *Anopheles maculipennis* complex GB2013 (1); *An. maculipennis* s.l. BTLHVDV2014 (1)	1.57	4.19	1.33	BOLD:AAA9632 (*Anopheles maculipennis*)
		*Anopheles daciae*	4					
BOLD: ABZ7976	42	*Culex laticinctus*	2	*Culex laticinctus* (27)	0.50	1.36	1.76	BOLD:AAA4752 (*Culex theileri*)
BOLD: ACB9122	80	*Aedes sticticus*	21	*Aedes sticticus* (57)*; Ae. rusticus* (1); *Ae. rossicus* (1)	0.31	1.28	1.70	BOLD:ACO9361 (*Aedes nigrinus*)
BOLD: AEG2154	21	*Aedes geniculatus*	7	*Aedes geniculatus* (20); *Aedes* sp. GW76 (1)	0.32	1.32	1.10	BOLD:AAM5898 (*Aedes geniculatus*)
BOLD: AEV9902	446	*Culex torrentium*	17	*Culex torrentium* (441); *Cx. pipiens* (2); *Cx. modestus* (1); *Aedes japonicus* (1)	0.37	2.74	1.04	BOLD:AAA4751 (*Culex pipiens*)

The largest number of species (28) was sampled from the Pannonian-Peripannonian region, followed by the Mediterranean region (19) and the mountainous region (18). Ten species were recorded in all three regions (Additional file 1: Table S1). One individual from the Intrudens Group (CROCU102-21) was recorded in the Pannonian-Peripannonian region (Klokočevci – Fig. 1) and represents new data not only for this region, but also for the Croatian fauna in general.

The success rate of COI amplification and sequencing from both directions in relation to the number of samples processed was 72.7% (197 / 271 specimens), while the percentage of successful amplification reactions of ITS2 was 98.5% (132 / 134 specimens of the *Anopheles maculipennis* complex). The remaining specimens were excluded from further analyses due to failed amplification or low sequence quality. A minimum of one and a maximum of 21 COI sequences were obtained per species, with an average of six.

**Fig. 2:**
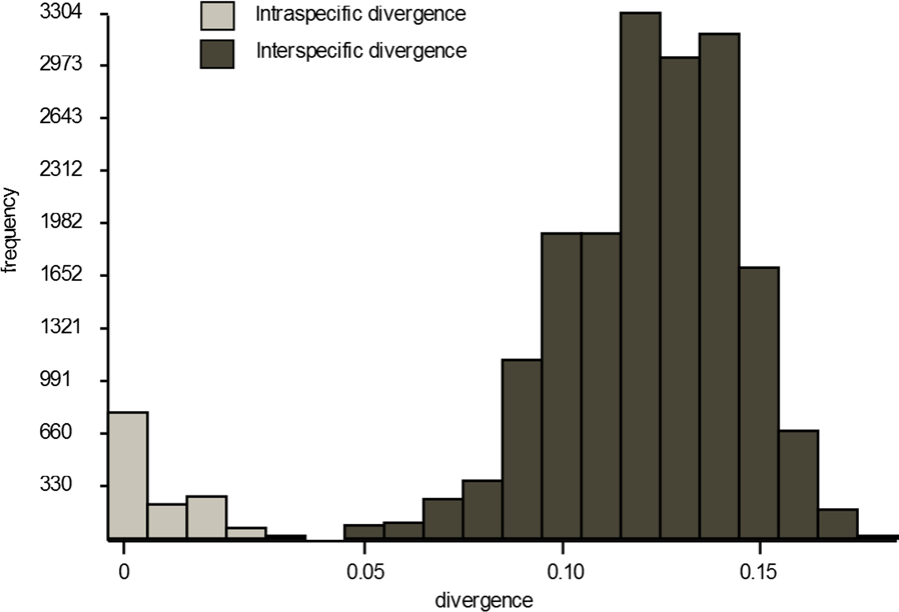
Frequency histogram of p-distances within and between investigated mosquito species (MOTUs) for the public BOLD dataset DS-CROCU2

The BIN-RESL algorithm in BOLD assigned the specimens to 31 BINs (Table 1), with most delineated MOTUs matching the morphological identification. Several BINs were discordant, with two or more species placed together. BIN discordance was present in the genus *Aedes* and the genus *Anopheles*, mainly in the species that were difficult to distinguish morphologically. These are the BINs with samples identified as *Ae. annulipes*, *Ae. cantans, Ae. riparius* and *Ae. excrucians* (BOLD:AAB1098); *Ae. rossicus* and *Ae. cinereus* (BOLD:AAP8897); *Ae. caspius* and *Ae. zammitii* (BOLD:AAB7911); *An. messeae* and *An. daciae* (BOLD:ABY8239). Barcode gap analysis in BOLD confirmed the presence of a clear barcoding gap within the public BOLD dataset DS-CROCU2 (Fig. 2). The concordance with the morphological identification was 98.48%.

In 13 species, the genetic distances of the species BIN to the nearest neighbour (NN) (i.e. non-specific specimen with the lowest interspecific distance in BOLD) were higher than the maximum intraspecific genetic distances, while other species had a lower value (Table 1). The mean intraspecific p-distance ranged from 0% to 1.68%, calculated for MOTUs delimited with BIN-RESL. The largest intraspecific divergence was observed in BIN BOLD:AAA7067 for the species *Ae. vexans* (7.22%), followed by BIN BOLD:AAB2483 for *An. hyrcanus* (5.32%) and BIN BOLD:AAA4751 for *Cx. pipiens* s.l. (5.02%). Only one species, *Or. pulcripalpis* (BOLD:AAW9535), showed no intraspecific variability in BOLD (Table 1).

**Fig. 3:**
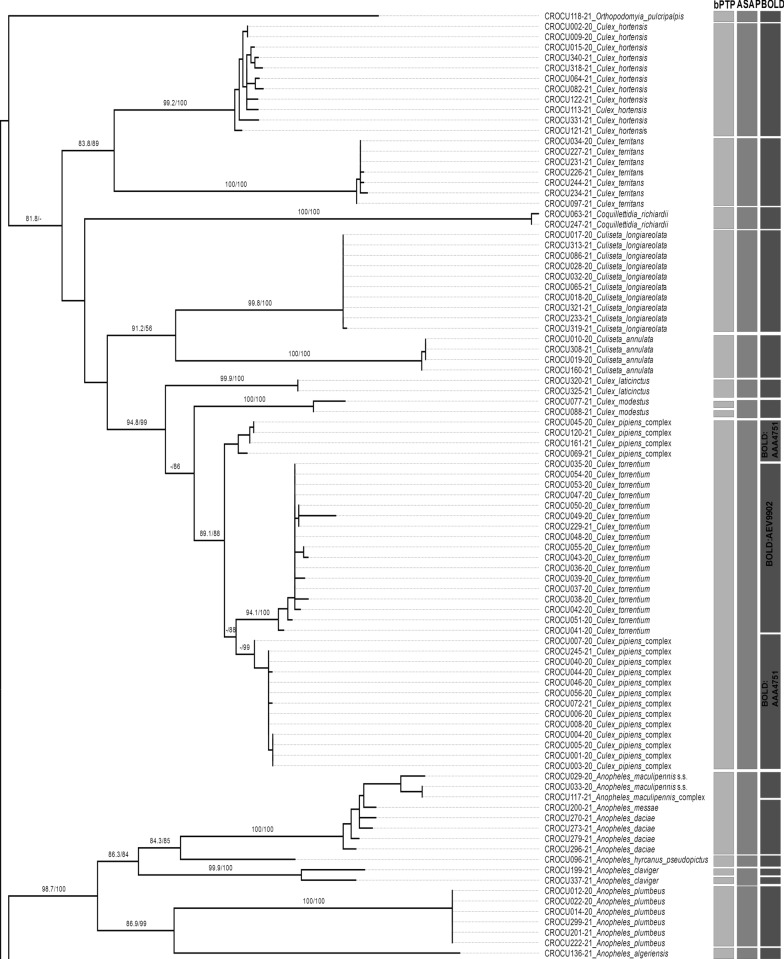

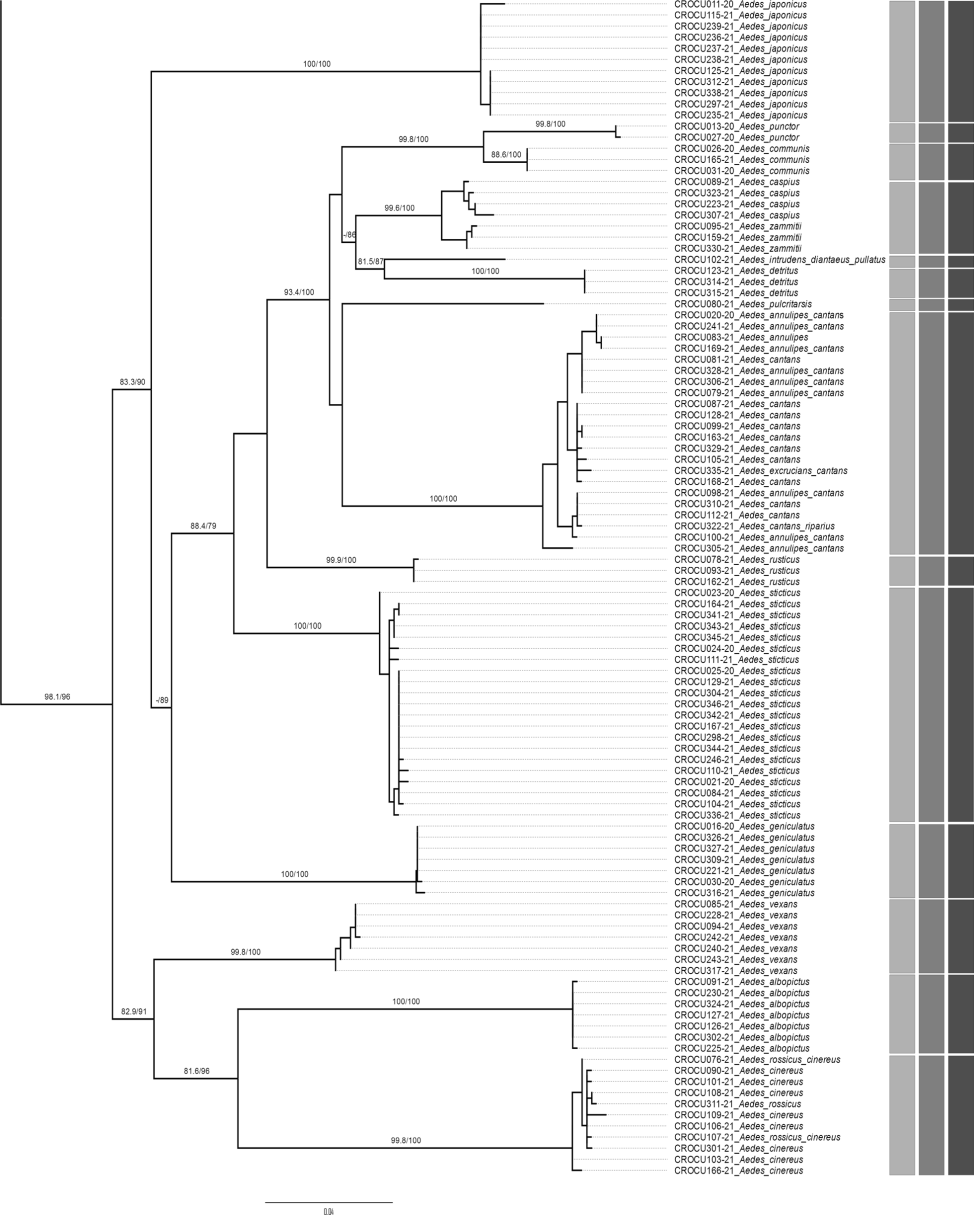
ML tree based on COI DNA sequences of the specimens sequenced in this study. Numbers on branches denote bootstrap / aLRT support values (values lower than 70% are not shown). The results of species delimitation methods (bPTP, ASAP and BIN-RESL) are shown as vertical bars on the right

The ASAP method delimited 28 MOTUs (Fig. 3). Some species of the genera *Culex, Aedes* and *Anopheles* were assigned to the same MOTUs, mostly again the species that are difficult to distinguish morphologically (Fig. 3). A very similar delimitation was achieved using the bPTP method, resulting in 30 MOTUs being assigned to our dataset. Using bPTP and BIN-RESL analysis, *An. claviger* s.s. was subdivided into two MOTUs. Also, *Cx. modestus* was subdivided into two MOTUs, but only using the bPTP delimitation method. The BIN-RESL method divided *Cx. torrentium* and *Cx. pipiens* s.l. into two different BINs, while ASAP and bPTP analyses classified them to the same MOTU.

Most of the morphologically identified species formed well-supported monophyletic groups in the ML tree, which mostly corresponded to the assigned BINs, with support values between 81 and 100% bootstrap / > 0.9 aLRT (Fig. 3). Several species appeared to be paraphyletic, including *Cx. pipiens* s.l. in relation to *Cx. torrentium* and *An. daciae* and *An. messeae* in relation to *An. maculipennis* s.s.

Of the 132 specimens morphologically identified as *Anopheles maculipennis* complex, three species were confirmed using the ITS2 sequence: *An. maculipennis* s.s., *An. messeae* and *An. daciae*. *An. maculipennis* s.s. was the predominant species with 56.06% of the samples, followed by *An. messeae* with 36.36% and *An. daciae* with 7.57% of the samples.

Based on both morphological and molecular identification, a total of 34 species were identified in this study (Table 1).

## Discussion

This work represents the first comprehensive study aimed at generating DNA barcodes for the establishment of a DNA barcode library for the Croatian mosquito fauna. Based on morphological characteristics, 30 mosquito species were identified, divided into six genera, and four more species were confirmed based on molecular data, which in total represents about 63.5% of the total number of mosquito species recorded in Croatia [11]. The validity and accuracy of the data obtained was examined using species delimitation algorithms, with various clustering methods additionally supporting the MOTUs obtained. This study shows that, in addition to morphological classification, molecular taxonomy can also be a suitable tool for the identification and delimitation of mosquito species in Croatia, as has recently been shown for many other countries [36–38, 57, 62, 78, 79]. The concordance of morphological and molecular identification in this study is high, which is also consistent with recent similar studies [38, 62, 80]. Morphological and molecular data discrepancies might be due to misidentifications, resulting in inaccurate species names for sequences in public databases [81]. This study found minimal differences in species delimitation methods, mostly for morphologically indistinguishable species. The effectiveness of barcoding was confirmed by the presence of a clear barcoding gap, which is necessary for the effective application of DNA barcodes to identify specimens and delimit species [62, 82]. According to Meyer & Paulay [83], a barcoding gap can be defined as the difference between the mean intraspecific sequence variability and the interspecific variability for congeneric COI sequences.

Based on DNA barcode analysis, most of the species morphologically identified in this study were categorised into established groups in ML tree analysis. A few individuals in certain taxa of the genera *Aedes* and *Anopheles*, such as *Ae. annulipes*/*cantans*/*excrucians*, *Ae. rossicus*/*cinereus, Ae. caspius/zammitii,* and *An. daciae*/*messeae*, were grouped within the same BINs, suggesting that the COI gene may not be informative enough for distinguishing among these species. This is also confirmed by the fact that these taxa occur in similar combinations in BOLD BINs. According to recent studies, these taxa are phylogenetically very closely related, although they have different morphological characteristics [38, 57, 62].

The *Anopheles maculipennis* complex consists of several species, seven of which have been recorded to belong to the Croatian fauna [11]. In this study three of them were identified, namely *An. maculipennis* s.s., *An. messeae* and *An. daciae*. In the COI sequence-based species delimitation, the *Anopheles maculipennis* complex was divided into two different MOTUs, *An. maculipennis* s. s. and *An. messeae/daciae,* by the results of the BIN-RESL algorithm. These two BINs are mutually nearest neighbours in BOLD, with only 1.33% distance between them, while in both BINs the highest intraspecific distance is about 4% (Table 1). The other two delimitation methods do not support this partitioning, as they group all *An. maculipennis/messeae/daciae* specimens into a single MOTU, with *An. messeae/daciae* specimens appearing as a paraphyletic group in the ML tree with respect to the *An. maculipennis* s.s. samples. This confirms the highly entangled situation in this species group which requires further investigation involving other molecular markers, similar as it was observed in Sedaghat et al. [84].

A rare species within the *Anopheles maculipennis* complex, *Anopheles melanoon*, previously recorded in the Croatian areas of southern Dalmatia and Istria [85], could not be confirmed in this study, possibly due to environmental modification of its distribution area. The occurrence of this species is closely linked to areas with horse and cow stables [86, 87], and such habitats have become quite rare. Other species within this complex previously detected in Croatia (*An. atroparvus*, *An. sacharovi* and *An. labranchiae*) [88, 89] were also not found in other recent surveys [50, 85]. Future studies should definitely focus on the detection of these species to confirm or exclude their current distribution in Croatia, as they are the most important malaria vectors in Europe [30].

The sibling species *Cx. torrentium* and *Cx. pipiens* s.l. can only be distinguished morphologically based on the characteristics of the male genitalia [67, 90]. The results of this study are consistent with the results of studies in Belgium [57] and show that these two species are separated as distinct MOTUs based on the BIN-RESL method, which was not the case in another recent study [62].

In contrast, the ASAP and bPTP methods did not show sufficient discrimination between species within genetically closely related species groups such as the *Culex pipiens* complex or the *Anopheles maculipennis* complex. For each of these two groups, the species are grouped into a single MOTU according to ASAP and bPTP.

The two sibling species *Anopheles claviger* s.s. (Meigen) and *Anopheles petragnani* Del Vecchio belong to the *Anopheles claviger* species complex [67], but *An. petragnani* has not yet been recorded in the Croatian fauna [11]. Within this species complex, COI has proven to be a sufficient tool to distinguish the two species [62]. Our two *An. claviger* s.s. specimens formed a strongly supported clade in the ML tree according to ASAP method, but were identified as separate MOTUs using bPTP and BIN-RESL methods. The specimen CROCU199-21 from a mountainous region was grouped in a BIN BOLD:AAM4220, where a wide European distribution with larger genetic distance (max. 3.56%) is observed. Another specimen, CROCU077-21 from the Mediterranean region, was grouped in BIN BOLD:AAE3979, with a narrower distribution (mainly Iran and Kosovo) and somewhat lower distances (max. 3.15%) (Table 1). A third BIN in BOLD with specimens from Tajikistan and China suggests that the species, *An. claviger* s.s., may be divided into several cryptic species. However, *An. petragnani* is placed in a separate BIN, including specimens from western Europe, some of them misidentified as *An. claviger*.

*Culex modestus* is considereda potential vector of WNV [91]. In this study, two individuals of *Cx. modestus* were detected in the Pannonian-Peripannonian region of Croatia near wetlands. This species is considered as the main vector of WNV in similar areas of southern France [92]. Although its dispersal ability is low, it could serve as an important enzootic and, given its ornithophilic and mammalophilic biting behaviour, bridging vector in natural/rural wetlands across Europe [67]. Therefore, in order to gain insights into the genetic structure of populations in this region, it should be studied in more detail in the future, using a larger number of individuals and localities.

Currently, there is no comprehensive database for all European mosquito species of the subgenus *Ochlerotatus*. According to Becker et al. [67], species groups associated with European species were classified according to region (Palaearctic, Fennoscandia, Germany and former USSR), which are also mentioned in our study (see below). The differences in the classification of species within each region will remain as long as there is no global analysis of the subgenus [67].

Within the Caspius Group there are six species, *Aedes berlandi*, *Ae. caspius*, *Aedes dorsalis*, *Aedes mariae*, *Ae. pulcritarsis* and *Ae. zammitii* [67], of which only *Ae. berlandi* has never been recorded in Croatia. According to all species delimitation algorithms used in this study, *Ae. caspius* and *Ae. zammitii* are grouped in the same MOTU, although they can be clearly distinguished morphologically. A similar case was observed in a recent study on the territory of Spain [80] for *Ae. mariae* and *Ae. caspius* which were grouped in the same MOTU. In our study the minimum interspecific distance between *Ae. zammitii* and *Ae. caspius* was 1.15%, similar to that in the aforementioned study [80], which is value lower than values typical between species [33]. Clearly, additional analyses of other loci that may better support species delimitation are required for a more accurate separation of species within this group.

Of the Annulipes Group, *Ae. annulipes, Aedes behningi, Ae. cantans, Ae. excrucians, Aedes flavescens* and *Ae. riparius* were recorded in Croatia [11, 67]. In general, distinction between species *Ae. annulipes* and *Ae.cantans* cannot always be accomplished with certainty based on morphological characters. Some sequences of the specimens morphologically identified as *Ae. annulipes*, *Ae. cantans* or *Ae. excrucians* matched the sequences in BIN: BOLD:AAB1098, which includes samples identified as *Ae. annulipes/cantans* or *Ae. excrucians/cantans*. One specimen, which had been assigned to the species *Ae. behningi* according to morphological characteristics, turned out to be *Ae. cantans* based on molecular analysis. In our ML tree, all specimens morphologically assigned to *Ae. annulipes* and *Ae. cantans* clustered into a single, well-supported clade, and all three species delimitation algorithms grouped the samples into a single MOTU. Consequently, COI proved to be an insufficient marker for distinguishing species within this species, as previously mentioned in other studies [38, 57].

In this study, the species *Ae. cinereus* and *Ae. rossicus* are grouped in the same MOTU according to all methods of species delimitation. Some samples morphologically identified as *Ae. cinereus* or *Ae. rossicus* were also confirmed based on COI sequences, while some could no be differentiated and had to be labelled as *Ae. rossicus/cinereus* (Fig. 3, Table 1). All individuals were collected in the Pannonian-Peripannonian region of Croatia. The taxonomic status of the species of the subgenus *Aedes* occurring in the Palaearctic is still unclear. *Ae. cinereus, Ae. rossicus* and *Aedes esoensis* were considered subspecies of the nominate form *Ae. cinereus* by Gutsevich et al. [93]. This view is not generally accepted, as *Ae. cinereus* and *Ae. rossicus* overlap strongly in Europe, the larvae often occur at the same breeding sites (which was also the case for our samples CROCU107-21, 108–21 and 109–21 from the same location and habitat—Fig. 3, Table 1), and transitional forms are unknown [67]. This is also supported by the fact that these two species are often found together, with samples from this study being assigned to the same BIN (BOLD:AAP8897).

A possible new species for the Croatian fauna, detected in this work, belongs to the Intrudens Group, which includes the species *Ae. diantaeus*, *Ae. intrudens* and *Ae. pullatus* [67]. Morphologically, the respective sample, CROCU102-21, was incorrectly determined as *Aedes punctor*, but after BIN-RESL analysis it was placed in BIN BOLD:AAF2904, which includes samples mainly identified as *Ae. intrudens* and *Ae. diantaeus*. This sequence matches 99.06% with the sequence of a specimen from Russia (KC855601) and 98.96% with a specimen from Sweden (JX040505), both of which were identified as *Ae. intrudens*. According to our photographs (available in the BOLD dataset DS-CROCU2) and the identification key according to Becker et al. [67], our specimen also corresponded to the description of the species *Ae. intrudens.* The eggs of this species overwinter, and the larvae are found from early spring onwards until the beginning of summer [67], which is consistent with our finding in April. In addition, the larvae of this species develop in temporary forest pools with dead leaves on the bottom [67], supporting our observations of its habitat. Considering that one single specimen was found, future studies should focus on the rediscovery and morphological and molecular confirmation of this species so that it can be reliably included in the list of Croatian mosquito species.

This six-year study covered 63.46% of the total mosquito fauna in Croatia. The distribution of species in Croatia by region determined in this study essentially corresponds to the results of previous studies [11, 50, 51]. It should be emphasized that some species included in the currently valid Croatian mosquito species list [11] were last recorded a long time ago, such as species of the *Anopheles maculipennis* complex: *An. atroparvus*, *An. sacharovi* and *An. labranchiae* [88, 89, 94]. Efforts have been made to eliminate malaria mosquitoes over the past centuries, and habitat changes and pollution may have contributed to reducing their population or causing extinction in this area [95–97]. However, as these species are the main vectors of malaria in Europe [30], future research should focus on confirming their presence (or absence) in the area. Species that were not recorded in this study but had been recorded in some other recent studies [98–100] are monocyclic, rare or uncommon [67], such as *Aedes cataphylla*, *Aedes leucomelas*, *Ae. behningi*, *Ae. riparius*, *Culex martinii*, *Culiseta morsitans* and *Culiseta subochrea*. In addition, some species are linked exclusively to certain habitats and periods of occurrence, and there was a high probability that they would not be recorded in this type of research. It is necessary to focus on these rare and unrecorded species in the future so that they can be sampled specifically and thus expand the database of Croatian mosquito barcodes in BOLD.

## Conclusions

With barcoding sequences for 34 Culicidae species, the results of this study represent the basis for the establishment of a reference DNA barcode library for mosquitoes in Croatia. It has been proven that barcoding is an appropriate tool for the additional identification and delimitation of species of the Croatian mosquito fauna, even for closely related species such as *Cx. pipiens* s.l. and *Cx. torrentium*, while ITS2 proved a suitable marker to differentiate closely related species within the *Anopheles maculipennis* complex. The inability of COI to distinguish certain biotypes or other closely related species (such as *Cx. pipiens* biotype *molestus*; *Ae. annulipes/cantans; An. messeae/ daciae*) needs to be compensated for by additional molecular markers. In the future, studies should focus on confirming the species from the Croatian mosquito fauna list that were not included in this study. For the discovered potentially new species of the Intrudens Group, targeted investigations involving sampling of individuals in all life stages are needed to substantiate their inclusion in the list of Croatian mosquito fauna. Rapid and accurate identification is a crucial step in mosquito surveillance and control, so the data here are important not only for the assessment of biodiversity and of the geographical distribution of potential vector species in the studied area. The reference barcode sequences will contribute to future research on the mosquito fauna in Croatia, neighbouring countries and Europe in general, facilitate the identification or detection of potentially misidentified or cryptic species and provide a basis for invasive and vector species surveillance and monitoring projects.

### Supplementary Information

Supplementary Material 1: Table S1. All samples analysed in this study with their BOLD IDs, divided by regions of Croatia where they were found.

Supplementary Material 2. Dataset S1. Nucleotide alignments of COI and ITS2 datasets for all analysed specimens.

## Data Availability

All relevant data are within the manuscript and its additional files. All information about barcoded specimens can be found in the public BOLD dataset DS-CROCU2. The vouchers of barcoded specimens are kept in Josip Juraj Strossmayer University of Osijek, Department of Biology, and in Andrija Štampar Teaching Institute of Public Health in Zagreb. DNAs of barcoded specimens are kept in Josip Juraj Strossmayer University of Osijek, Department of Biology.
